# Effective and Generalizable Graph-Based Clustering for Faces in the Wild

**DOI:** 10.1155/2019/6065056

**Published:** 2019-12-14

**Authors:** Leonardo Chang, Airel Pérez-Suárez, Miguel González-Mendoza

**Affiliations:** ^1^Tecnologico de Monterrey, School of Engineering and Science, Monterrey, Mexico; ^2^Advanced Technologies Application Center, CENATAV, Havana, Cuba

## Abstract

Face clustering is the task of grouping unlabeled face images according to individual identities. Several applications require this type of clustering, for instance, social media, law enforcement, and surveillance applications. In this paper, we propose an effective graph-based method for clustering faces in the wild. The proposed algorithm does not require prior knowledge of the data. This fact increases the pertinence of the proposed method near to market solutions. The experiments conducted on four well-known datasets showed that our proposal achieves state-of-the-art results, regarding the clustering performance, also showing stability for different values of the input parameter. Moreover, in these experiments, it is shown that our proposal discovers a number of identities closer to the real number existing in the data.

## 1. Introduction

With the broad establishment in recent years of video surveillance systems and the billions of cameras embedded in smartphones, face analysis from images is an increasingly prevalent task for government agencies and industry alike. While face analysis has been an active research area for several decades, most of the prior work was focused on face verification/identification in relatively constrained environments (e.g., near-frontal poses and under controlled lighting conditions) [1, 2].

**Figure 1:**
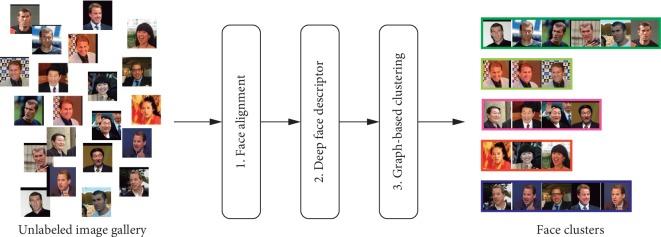
Given an unlabeled face image gallery, face clustering is performed by (1) aligning faces, (2) computing a face representation for each face, and (3) performing feature clustering in this representation space.

Less studied is the problem of clustering faces in unconstrained environments. Face clustering is the task of grouping unlabeled face images according to the individual identities present in the data.  Figure 1 shows an overview of the face clustering workflow followed in this paper. Several challenges must be confronted for clustering faces captured in unconstrained scenarios. In surveillance applications, the quality of available face images is typically quite low, presenting arbitrary poses, illumination changes, occlusions, and low resolution.

In this paper, we propose an effective graph-based method for clustering faces in the wild. For obtaining face representations, we use a deep convolutional network, specifically a 29-layer ResNet (from Residual Neural Network), which produces a 128-dimensional face descriptor. For clustering face descriptors, we propose to use a graph-based clustering algorithm whose result is later processed in order to join homogeneous clusters that could have been divided in the clustering step, caused by the sparseness of the input graph.

The main contribution of our proposal is that no assumption about the data is used. Only a threshold parameter is required to build the initial face graph. Regarding this threshold, our experiments on four well-known face datasets showed that it is possible to recommend a single threshold value that will provide clustering results closer to the best possible results. This is a significant advantage of the proposed method since, in real applications, usually there are no labeled data where parameters can be trained. Also, the number of identities discovered by our proposal was closer to the ground truth number of identities compared to those discovered by the other evaluated algorithms, achieving state-of-the-art clustering results, under several evaluation measures.

The rest of the paper is organized as follows: Section 2 describes some relevant and previous work on face clustering and graph-based clustering. Later, in Section 3, our method for clustering faces is detailed. Experimental evaluation and comparison of our method with state-of-the-art algorithms are reported in Section 4.  Section 5 concludes the paper.

## 2. Related Work

### 2.1. Face Clustering

Face clustering is the task of grouping faces by their underlying identity. It is closely related to the face recognition problem but has several fundamental differences. In face recognition, the goal is to verify (1 : 1 comparison against an enrollment face image) or find (1 : N comparisons against a face gallery) the identity of a given subject, assuming that the identity of subjects in the gallery/enrollment is known beforehand. Therefore, face recognition could be considered a supervised classification task. In contrast, face clustering is considered an unsupervised classification problem since no labeled data are provided. There are some works in face clustering considered as semisupervised clustering, where several constraints, mainly from videos, can be converted into must-link and cannot-link constraints and later used to improve face clustering [3, 4, 5]. While a large body of work has been conducted on both face recognition and data clustering in general, the challenging problem of face clustering is a less studied topic, especially when dealing with a large number of images and subjects, and also for unconstrained scenarios.

Cao et al. [6] developed a tensor clustering algorithm for face images, which can handle the faces with different expressions, illuminations, block occlusions, random pixel corruptions, and various disguises. Their method firstly finds a lower-rank approximation of the original tensor data using an L1-norm optimization function. Then, they compute the high-order singular value decomposition of the approximate tensor to obtain the final clustering results. The authors formulate the process of approximation into a framework of tensor principal component analysis with L1-norm.

Otto et al. [7] developed a version of the rank-order clustering algorithm of Zhu et al. [8], leveraging an approximate nearest neighbor method for improved scalability and simplifying the actual clustering procedure to achieve improved scalability and clustering performance. The authors evaluated large-scale clustering performance by combining the Labeled Faces in the Wild (LFW) dataset with up to 123 million of unlabeled images (downloaded from the web) and clustering the augmented dataset. Also, clustering results on video frames leveraging the YouTube Faces (YTF) database are presented.

Shi et al. [4] proposed a face clustering method, called Conditional Pairwise Clustering (ConPaC) to group a face collection according to the subject identity. ConPaC uses a direct estimation of an adjacency matrix derived from pairwise similarities between faces, which are computed over a learned deep residual network representation. The method is also extended to the semisupervised clustering by accepting a set of pairwise constraints (either must-link or cannot-link assignments) on the similarity matrix. The evaluation was performed on two unconstrained face datasets, i.e., LFW and IJB-B.

Shi et al. [9] proposed a self-learning framework for face clustering, which consists of two major stages: image decorrelation and self-paced learning. The authors extended the two-dimensional whitening reconstruction [10] to handle local image patches in order to reduce image redundancy while preserving significant local features. Then, the authors group the semantically similar faces by using a self-paced learning model, which is inspired by the following observations: the learning process of humans goes from easy to complex tasks, the prior knowledge of human might change with the increase in learned experience and more prior knowledge usually leads to better prediction accuracy. The method proposed in [9] was evaluated in controlled environments in a subset of the Extended Yale-B [11] and AR [12] databases and in unconstrained environments in a subset of the LFW [13].

### 2.2. Graph-Based Clustering

Clustering is a fundamental technique in pattern recognition and data mining which aims to organize a set of objects into a set of classes called clusters. The idea is that objects belonging to the same cluster are similar enough to infer they are of the same type, while objects belonging to different clusters are different enough to assume they are of different types [14].

Many clustering algorithms have been proposed so far: *k*-means, single link, CURE (meaning Clustering Using Representatives), DBSCAN (meaning Density-Based Spatial Clustering of Applications with Noise), and Expectation Maximization are well-known examples; see [15]. Several clustering algorithms have been successfully applied in contexts like information retrieval [16], bioinformatics [17], medicine [18], image segmentation [19], and cybersecurity [20], among others.

An important class of clustering algorithms is graph-based clustering algorithms. These algorithms represent the collection of objects as a graph *G*=〈*V*, *E*〉, where *V* is the set of vertices, i.e., objects of the problem at hand, and *E* is the set of edges, and each edge represents the (dis)similarity relationship existing between a pair of objects. *G* could be directed or undirected depending on the function used for computing the (dis)similarity between the vertices. *G* also can be weighted or unweighted; in the first case, the weight of an edge *e* ∈ *E* is denoted as *w*_*e*_.

Graph-based algorithms provide a simple way to represent both the objects and the relations among them. Also, they do not impose any restriction to the representation space of the objects or the (dis)similarity measure between objects; these characteristics increase their practical usefulness. Usually, graph-based algorithms build the clustering through a covering of the graph *G*, using a special kind of subgraph [21]. In this context, each subgraph, or a modification of it, is assumed to be a cluster. Nevertheless, there are also graph-based algorithms which use other approaches like optimization [22] or game theory [23], among others.

Since, in this work, we are addressing the clustering of face images and taking into account that we do not want images from different subjects to share a cluster, we focus on producing a disjoint clustering. Nevertheless, other types of clustering are reported in the literature, such as overlapping and fuzzy clustering.

### 2.3. Clustering Evaluation

The notion of Clustering Evaluation Measure or Clustering Validity Index emerged as an answer to the necessity of selecting, from a set of clustering algorithms and a given dataset, the one having the best performance. It is expected that these validation measures can be impartial and do not have any preferences on any particular clustering algorithm. The evaluation measures reported so far can be classified as *external* or *internal* [14].

External measures evaluate a clustering solution based on how much this solution resembles a given set of classes, commonly known as *ground truth*, which has been manually labeled by human experts. On the contrary, internal measures rely only on the information in the clusters, and they validate the solutions taking into account the accomplishment of one or more properties, which are implicitly or explicitly measured by the index. From these two kinds of measures, the most widely used are the external measures.

Several external measures have been reported in the literature: entropy [24], Jaccard coefficient [25], and V-measure [26], among others; these measures are different according to their mathematical foundations, biases, and limitations. Also, several works have analyzed which properties or mathematical constraints should be fulfilled by an evaluation measure [26–29]. In fact, the work of Amigo et al. [27] proposes four formal constraints which cover most of the previously reported ones.

For evaluating the face clustering algorithm proposed in this work, we employ the same measures used in [4]; that is, we use the Pairwise Fmeasure [4] and the BCubed Fmeasure [27]. The Pairwise Fmeasure, denoted as F-measure, is an index commonly used in the literature for evaluating clustering results, which in turn fulfills two of the four constraints introduced in [27]: *cluster homogeneity* and *cluster completeness*. This measure is defined as follows:

(1) $$\begin{array}{c} \text{F}‐\text{measure}=\frac{2\cdot \text{PW}\_\text{Precision}\cdot \text{PW}\_\text{Recall}}{\text{PW}\_\text{Precision}+\text{PW}\_\text{Recall}}, \end{array}$$

where PW_Precision and PW_Recall denote the pairwise precision and pairwise recall, respectively, and they are defined as follows:

(2) $$\begin{array}{c} \text{PW}\_\text{Precision}=\frac{T_{11}}{T_{11}+T_{10}},\\ \text{PW}\_\text{Recall}=\frac{T_{11}}{T_{11}+T_{01}}, \end{array}$$

where *T*_11_ are the number of pairs of objects (images in our case) which belong to the same cluster and to the same class, *T*_10_ is the number of pairs of objects belonging to the same cluster but in different classes, and *T*_01_ is the number of pairs of objects which belong to the same class but to different clusters.

On the contrary, given that F-measure has a bias to large clusters, we decided to use also the BCubed Fmeasure, denoted as FBcubed, which in turn meets the four constraints proposed in [27] and weights clusters linearly based on their size. This measure is defined as follows:

(3) $$\begin{array}{c} \text{FBcubed}=\frac{2\cdot \text{Bcubed}\_\text{Precision}\cdot \text{Bcubed}\_\text{Recall}}{\text{Bcubed}\_\text{Precision}+\text{Bcubed}\_\text{Recall}}, \end{array}$$

where Bcubed_Precision and Bcubed_Recall denote the Bcubed precision and Bcubed recall, respectively, and they are defined as follows:

(4) $$\begin{array}{c} \text{Bcubed}\_\text{Precision}=\frac{1}{N}\cdot \overset{N}{\underset{i=1}{\sum }}\underset{j\in C_{i}}{\sum }\frac{\text{Correctness}\left(i,j\right)}{\left|C_{i}\right|},\\ \text{Bcubed}\_\text{Recall}=\frac{1}{N}\cdot \overset{N}{\underset{i=1}{\sum }}\underset{j\in L_{i}}{\sum }\frac{\text{Correctness}\left(i,j\right)}{\left|L_{i}\right|}, \end{array}$$

where *N* is the number of objects, *C*_*i*_ and *L*_*i*_ refer to the *i*^th^ cluster and class, respectively, |·| refers to the size of the set, and Correctness(*i*, *j*) is 1 if the *i*^th^ and *j*^th^ objects belong both to the same cluster and to the same class; otherwise, its value is 0.

## 3. Proposed Face Clustering

### 3.1. Face Descriptor

**Figure 2:**
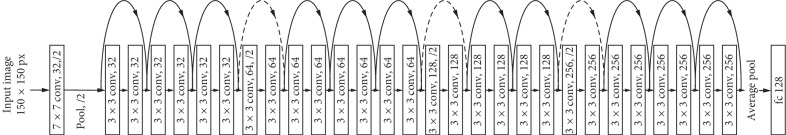
Network architecture of the ResNet-29 network used for face descriptor extraction. The dotted shortcuts increase dimensions. The input is a 150 × 150 image, and the output is a 128 floating-point values vector.

In recent years, deep convolutional neural networks have led to a series of breakthroughs for unconstrained face recognition, allowing us to deal with face images containing extreme poses, illumination, and resolution variations. Since we are interested in clustering faces in unconstrained scenarios, we use a face representation based on a deep convolutional neural network. The model is a ResNet network with 29 convolutional layers obtained by Davis [30]. It is a version of the ResNet-34 network proposed in [31] where five layers were removed, and the number of filters per layer was reduced by half, in order to improve the efficiency.  Figure 2 shows the network architecture of the ResNet network used.

**Figure 3:**
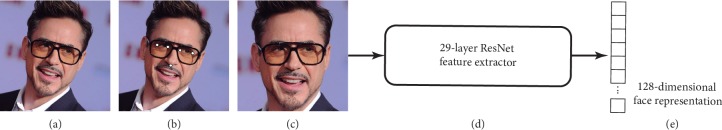
Face representation overview. Given a face image (a), five keypoints are detected (b) which are used to normalize the face image. The normalized image (c) serves as input for a ResNet network (d), and its 128-dimensional output (e) is used as face representation.

For a given input face image, five landmark points are detected (i.e., the corners of the eyes and the bottom of the nose) using the implementation provided in [30]. Using the detected points as reference, 2D face alignment is performed by using affine transformations to obtain a face of 150 × 150 pixels and 0.25 padding. The aligned image is used as input of the ResNet-29 network, and a 128-dimensional face descriptor is obtained.  Figure 3 shows an overview of the face representation process.

### 3.2. Clustering Method

Based on the advantages offered by graph-based algorithms and taking into account that we want to process a large number of images, we decided to adopt the Chinese Whispers approach [32] (CW, for short). CW is an efficient and effective algorithm for obtaining a partition of nodes from a weighted and undirected graph. In our case, the input graph *G* is built using the images, represented using the descriptor proposed in Section 3.1, as vertices and using the Euclidean distance for measuring the distance between two images. It is important to highlight that, in the computation of the input graph, we only consider those edges whose weights are less than a predefined threshold. Details and discussion about the impact of this threshold in the clustering results are provided in Section 4.3.

Intuitively, CW works as follows: First, it assigns a different class to each node in the graph. After that, the nodes are processed for a predefined number of iterations by assigning to each node the strongest class in their neighborhood. Let *v* be a vertex. The strongest class in the neighborhood of *v* is the class whose sum of edges weights to *v* is maximal among the edges to which *v* belongs to. In case of ties among classes, one of them is randomly selected.

A drawback of the CW algorithm is that it can produce a large number of clusters, depending on the sparseness of the input graph. We were able to verify this fact from preliminary experiments using experimental datasets. In fact, what is more concerning is that this drawback could make CW divide a homogeneous cluster into two or more clusters.

In order to overcome the aforementioned limitation of CW, we introduce a postprocessing phase, composed of two steps, which works as follows.

Let *C*={*C*_1_, *C*_2_,…, *C*_K_} be the set of clusters obtained by the CW algorithm. Let Min_*C*_*i*__ and Avg_*C*_*i*__, with *C*_*i*_ ∈ *C*, be the lowest and the average weight of the edges inside cluster *C*_*i*_, respectively. Let *W*_(*C*_*i*_, *C*_*j*_)_=∑*w*_*e*_ be the sum of the weights of all edges *e* connecting clusters *C*_*i*_ and *C*_*j*_, such that *w*_*e*_ ≥ Min_*C*_*i*__ or *w*_*e*_ ≥ Min_*C*_*j*__.

**Figure 4:**
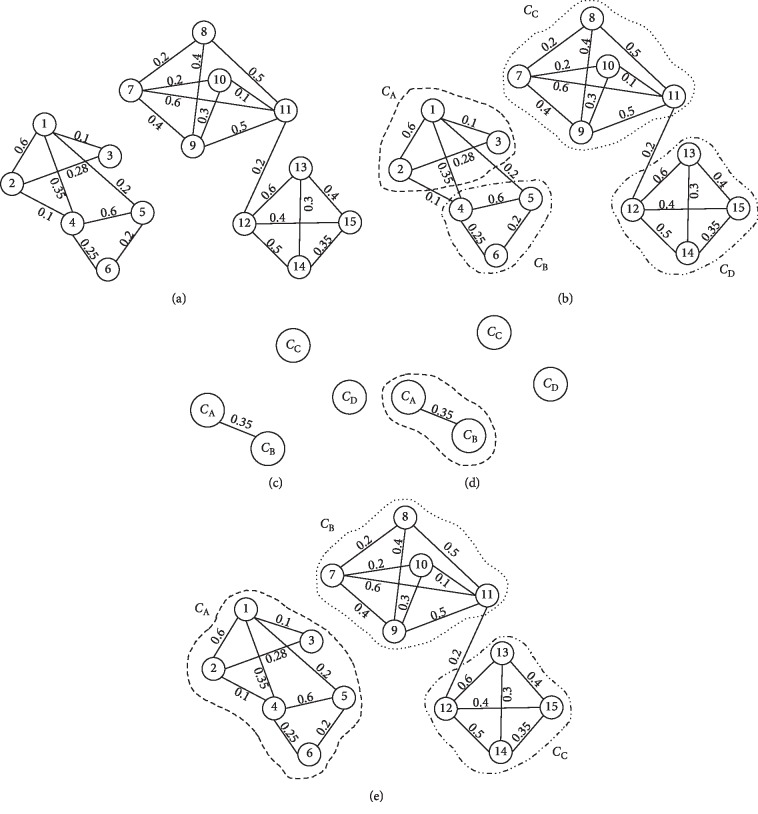
Proposed face clustering method overview. (a) Faces graph. Each vertex represents a face, and an edge is drawn between faces with distance less than a given threshold. (b) Initial clustering result. Each cluster is delimited by dotted lines. (c) Graph obtained by considering each CW cluster as a vertex and drawing an edge between two clusters if they are neighbors. (d) Clustering resulting from processing the graph in Figure 4(c), using the CW algorithm. (e) Final clustering obtained.

First, we build from *C* a graph *G*′=〈*V*′, *E*′〉 where each vertex *u* ∈ *V*′ is a cluster in *C* and there is an edge between two clusters if they are neighbors. Two clusters *C*_*i*_ and *C*_*j*_ are neighbors if *W*_(*C*_*i*_, *C*_*j*_)_ ≥ Avg_*C*_*i*__ or *W*_(*C*_*i*_, *C*_*j*_)_ ≥ Avg_*C*_*j*__. The intuitive idea behind the construction of this graph is to identify, using a different level of abstraction, those clusters highly related that could represent a single class divided into several parts (i.e., subclusters). Once *G*′ is built, it is processed using the same strategy of CW in order to build the final set of disjoint clusters.  Figure 4 shows a graphical overview of the main steps of the proposed method.

## 4. Experimental Evaluation

In this section, we present the overall evaluation and comparison of the proposed face clustering method. First, we describe the used datasets and evaluation protocols. Secondly, we evaluate the clustering performance of our proposal and compare it with related works in terms of clustering performance and computation time. Finally, we analyze the effect of threshold setting in the face clustering performance.

### 4.1. Datasets

**Figure 5:**
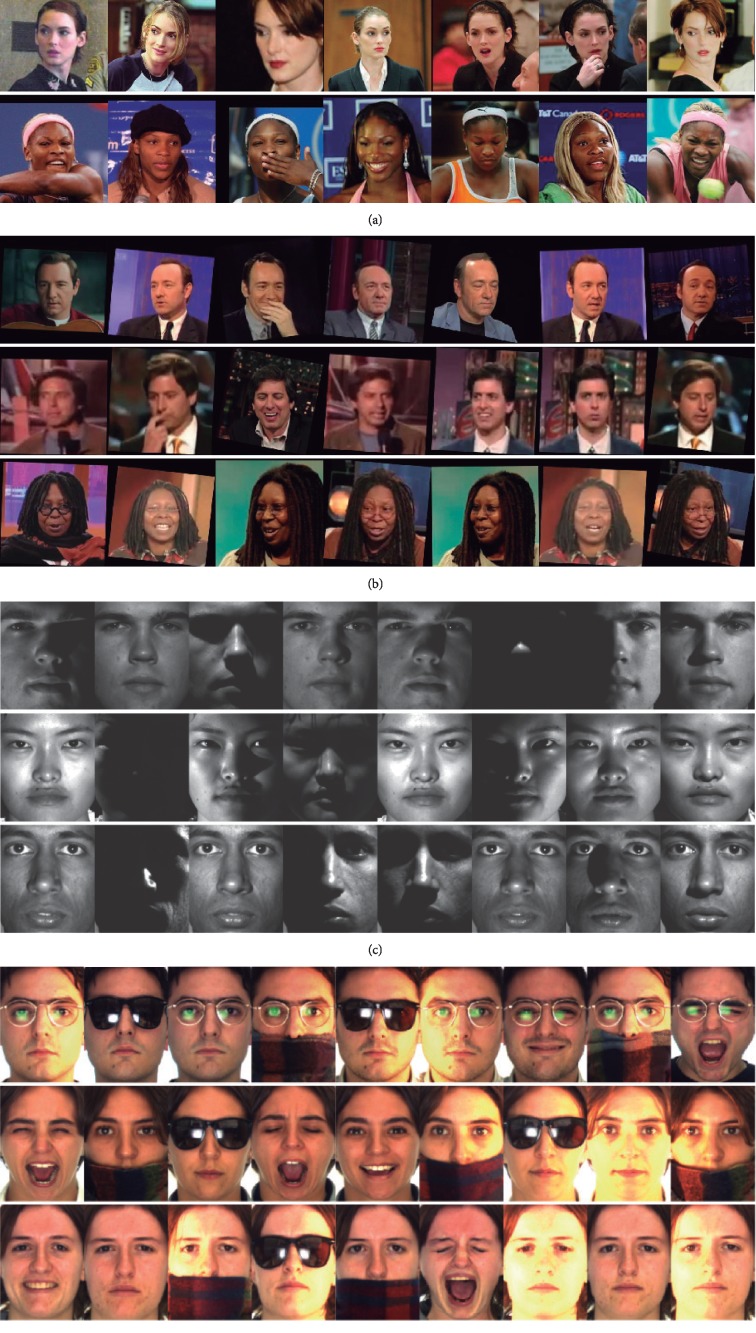
Example face images from the (a) LFW, (b) YTF, (c) Extended Yale-B, and (d) AR datasets. Large intra- and interdataset variations are present in the four datasets, e.g., illumination, pose, resolution, scale, background, and occlusion.

The experiments were conducted on four well-known face datasets: the Labeled Faces in the Wild [13], the YouTube Faces [33], the Extended Yale-B [11], and the AR [34] datasets. These datasets feature both controlled and noncontrolled environments, with a wide range of variations, i.e., variations on expression, illumination, pose, resolution, background, occlusions, and resolution.  Figure 5 shows some example images from the datasets used.

- The Labeled Faces in the Wild dataset (LFT) [13] was designed for studying the problem of unconstrained face recognition. The dataset contains 13,233 images of faces of celebrities and public figures, collected from the web. Each face is labeled with the name of the person pictured. There are 1,680 of the 5,749 people in the dataset who have two or more distinct photos. The face images present variations on expression, illumination, pose, resolution, and background.
- The YouTube Faces (YTF) database [33] is a large video dataset designed for unconstrained face verification in videos. Similar to LFW, the dataset consists of videos of celebrities and public figures. It contains 3,425 videos of 1,595 subjects with significant variations on expression, illumination, pose, resolution, and background. An average of 2.15 videos is available for each subject. The average length of a video clip is 181.3 frames. For clustering, faces in individual frames are used.
- The Extended Yale-B face database (Extended Yale Database B) [11] was designed to conduct experiments under severe illumination variations. It contains 38 subjects where images were captured under 9 different poses and 64 different illumination conditions. A subset containing the frontal face images under the 9 different illuminations is also provided. In this subset, all the images have been manually aligned and cropped to 168 × 192 pixels. In our experiments, this subset is used.
- The AR Face database (AR) [34] contains over 3,200 color images corresponding to 126 subjects (70 men and 56 women). Images in the AR feature frontal view faces with different facial expressions, illumination conditions, and occlusions (sunglasses and scarf). In this paper, we use the face crops used in [35] that include 2600 images of 50 subjects (25 males and 25 females), manually aligned and cropped to 120 × 165 pixels.

### 4.2. Clustering Evaluation

In this section, we evaluate the clustering performance of our proposal and compare it with other relevant approaches. As a baseline we consider *k*-means clustering with three different *k* values, i.e., the true number of subjects, the number of clusters obtained by our proposal, and the number of clusters obtained by the best-performing approach different than ours. Also, as a baseline, we consider the Global Logical-Combinatorial Clustering algorithm (GLC) [36], which have shown outstanding results in several applications and addressed the clustering problem from a graph theory point of view, as in our proposal.

We also compare the performance of our face clustering method with that reported by two recent face clustering approaches, i.e., Approximate Rank-order [7] and ConPaC [4]. The reported results for Approximate Rank-order and ConPaC were obtained from their corresponding papers [4, 7], where face representations different to that presented in Section 3.1 were used. In addition, we include results using the Approximate Rank-order algorithm with the face descriptor described in Section 3.1. This was not possible for the case of ConPaC because neither code nor executable of the algorithm was publicly available. The rest of the algorithms compared in Tables 1–4 use the representation described in Section 3.1.

For *k*-means, we used the C++ OpenCV implementation. For GLC, we used our own C++ implementation of the method. For the Approximate Rank-order algorithm, we used the Python implementation publicly available online (https://github.com/varun-suresh/Clustering). Since the clustering result of GLC, Approximate Rank-order, and our proposal depends on a given distance threshold parameter and given that there is not a known effective method to compute it, we evaluate all the algorithms at several values of this parameter and report the best results. Further analysis of the impact of this parameter in the face clustering result is provided in Section 4.3.

As it can be seen in Table 1, for the LFW dataset, the proposed algorithm performs better than competing algorithms for both evaluation measures. Our proposal also obtains a number of clusters that is closer to the true number of identities of the LFW dataset. On the contrary, when clustering the ResNet-29 face descriptors of the LFW, the proposed method outperforms the Approximate Rank-order [7] algorithm, what suggests that the obtained improvement resides in the proposed clustering strategy. Consequently, it would be interesting to evaluate whether using the face descriptors utilized by Otto et al. in [7] could improve the results of our proposed method with respect to those of Approximate Rank-order.

In addition, as it can be seen in Table 1, the *k*-means algorithm obtains the lowest results for both F-measure and FBcubed, in the LFW dataset. Given that LFW dataset is highly imbalanced [37] and most subjects have only a single image, this result is expected since *k*-means is not able to handle well-imbalanced data [4].

For the experiments conducted in the YTF dataset, our proposal also achieves the highest clustering performance, as it can be observed in Table 2. In this case, it is worth mentioning that clustering the ResNet-29 face descriptors with the Approximate Rank-order [7] algorithm outperforms the results reported in [7] when using Approximate Rank-order with their own face descriptors. This may suggest that the face descriptor employed in our work is more robust to the specific variations present in the YTF, which is a video dataset captured in uncontrolled environments. On the contrary, since the data are better balanced in the YTF, *k*-means attained results closer to the rest of the algorithms when compared to those obtained for the LFW (see Table 1). Similar behavior is observed in Tables 3 and 4 for the Extended Yale-B and the AR datasets, respectively.

As it can be seen in Tables 3 and 4, the proposed algorithm also performs better than competing algorithms for both evaluation measures, discovering a number of clusters that is closer to the true number of identities in both the Extended Yale-B and the AR datasets. These datasets were captured in controlled environments with extreme illumination variations and occlusions. Since the face descriptor described in Section 3.1 was not trained to deal with such extreme variations, the clustering results are lower, specifically for the AR dataset (see Table 4).

It is important to highlight that, as shown in Tables 1–4, our proposed face clustering method was able to discover the number of clusters (identities) with better clustering performance than the compared algorithms. Also, it achieved better clustering performance results for both evaluation measures, specifically for the FBcubed, which does not boost the performance of the results as it might be the case of the F-measure since it is based on pairs.

### 4.3. Threshold Impact Evaluation

As mentioned in Section 3.2, our proposed face clustering algorithm depends on a given distance threshold parameter to build the initial face graph. In the case of the Approximate Rank-order [7] algorithm, a distance threshold is also specified; it is the threshold on similarity to balance between the precision and recall rate for a particular dataset being clustered [7].

**Figure 6:**
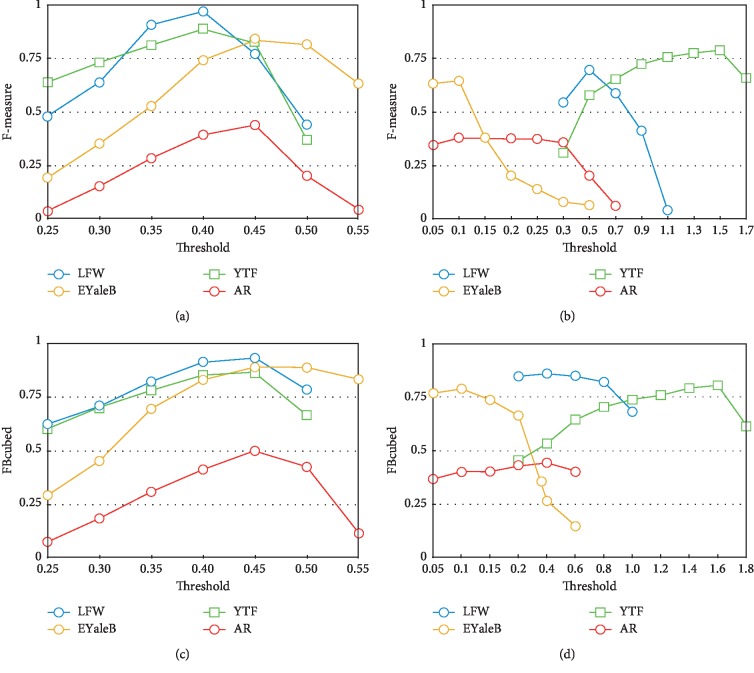
Clustering performance for different threshold values on the LFW, YTF, Extended Yale-B, and AR datasets obtained by (a) our proposal and (b) the Approximate Rank-order algorithm [7] evaluated using F-measure and performance obtained by (c) our proposal and (d) the Approximate Rank-order algorithm [7] evaluated using FBcubed.

In this section, we evaluate the impact of the distance threshold parameter in the face clustering result of our proposal across the four datasets. Also, we contrast these results with those obtained when varying the threshold parameters for the Approximate Rank-order algorithm [7]. Both algorithms were tested using several values of the threshold, and the results for the F-measure and FBcubed metrics are reported in Figure 6.

As it can be seen in Figure 6, the proposed face clustering method achieved its best clustering results for very similar threshold values, i.e., 0.40 and 0.45. This behavior is observed for the four datasets (i.e., LFW, YTF, EYaleB, and AR) and the two evaluation measures (i.e., F-measure and FBcubed). The same behavior was not observed for the Approximate Rank-order algorithm [7], where the best results were obtained for very different threshold values. It is worth noting that the four datasets used in the experiments have different conditions and characteristics, e.g., large interdataset variations of illumination, pose, resolution, scale, background, and occlusion; see Section 4.1. Therefore, it can be suggested that our proposed method is able to scale better for unseen data. In other words, when using our proposed algorithm on unseen data, threshold values between 0.40 and 0.45 are expected to obtain clustering results closer to its best possible results. However, for the Approximate Rank-order algorithm [7], it would be necessary to exhaustively test which threshold fits better for the new data. This is a significant advantage of the proposed method since in real applications, usually there are no labeled data where the threshold can be trained.

### 4.4. Computation Time Evaluation

In this section, we compare the computation time required to process each of the experimental datasets used in Section 4.2, by the algorithms analyzed in the previous sections. Since in [7], the Approximate Rank-order algorithm is evaluated using a different strategy for the extraction of face features and with the aim of focusing the analysis only on the computation time of clustering, all the clustering algorithms were tested with the same face feature descriptor, i.e., the ResNet-29 face descriptor introduced in Section 3.1. Using the same input data guarantees that the resulting time differences will be given only by the differences concerning the clustering algorithm.

Table 5 shows the computation time of each of the evaluated algorithms for clustering each of the experimental datasets; the shortest time for each dataset is highlighted.

As can be seen in Table 5, except for the YTF dataset, the clustering time of the proposed algorithm is close to the fastest algorithm, including *k*-means algorithm that has a simple clustering strategy. This fact shows that, in addition to achieving the best clustering performance, our proposal also presents computation times comparable to the rest of the state-of-the-art algorithms. Although the time for clustering YTF is worse than the rest of the algorithms, in that same dataset, our algorithm achieves significantly better results.

## 5. Conclusions

In this paper, for the problem of clustering faces in the wild, we have proposed an effective graph-based method which uses as face descriptor a ResNet-29 deep convolutional network. The proposed method outperforms several recent well-known clustering algorithms in the LFW, YTF, EYaleB, and AR datasets. The algorithm presented in this paper does not make any assumption about the face dataset to be clustered. Only a threshold parameter is required to build the initial face graph; nevertheless, in our experiments, we were able to find single threshold values in which clustering results are closer to the best possible results. Given that the four datasets used in our experiments were captured in different conditions and contexts, it can be suggested that the proposed method is able to scale better than the other compared methods. This is a significant advantage of the proposed face clustering method since, in many real applications, there are no labeled data available where parameters can be trained.

Our future work will include the exploration of incorporating pairwise constraints, i.e., must-link and cannot-link relations, in order to improve face clustering performance. This kind of constraint is very relevant for several applications, for example, faces tracked through a video sequence, semilabeled datasets, and others.

## Figures and Tables

**Table 1:** Comparison of clustering results in the Labeled Faces in the Wild (LFW) database.

Method	F-measure	FBcubed	Clusters
ResNet-29 + *k*-means (*k*=5749)	0.158	0.750	5749
ResNet-29 + *k*-means (*k*=5761)	0.153	0.749	5761
ResNet-29 + *k*-means (*k*=6352)	0.143	0.749	6352
ResNet-29 + GLC	0.920	0.911	6809
Approximate Rank-order [7]	0.870	—	6508
ConPaC [4]	0.965	0.922	6352
ResNet-29 + Approximate Rank-order	0.696	0.859	6564
ResNet-29 + ours (proposed)	**0.973**	**0.934**	5761

The true number of identities is 5,749, and the total number of face images is 13,233.

**Table 2:** Comparison of clustering results in the YouTube Faces (YTF) database.

Method	F-measure	FBcubed	Clusters
ResNet-29 + *k*-means (*k*=1595)	0.629	0.657	1595
ResNet-29 + *k*-means (*k*=1894)	0.595	0.656	1894
ResNet-29 + *k*-means (*k*=3050)	0.494	0.610	3050
ResNet-29 + GLC	0.832	0.787	21529
Approximate Rank-order [7]	0.71	—	—
ConPaC [4]	—	—	—
ResNet-29 + Approximate Rank-order	0.788	0.800	5563
ResNet-29 + ours (proposed)	**0.889**	**0.854**	3050

The true number of identities is 1,595, and the total number of face images is 621,126.

**Table 3:** Comparison of clustering results in the Extended Yale-B database.

Method	F-measure	FBcubed	Clusters
ResNet-29 + *k*-means (*k*=38)	0.653	0.703	38
ResNet-29 + *k*-means (*k*=42)	0.624	0.661	42
ResNet-29 + *k*-means (*k*=310)	0.250	0.271	310
ResNet-29 + GLC	0.737	0.787	310
Approximate Rank-order [7]	—	—	—
ConPaC [4]	—	—	—
ResNet-29 + Approximate Rank-order	0.646	0.788	125
ResNet-29 + ours (proposed)	**0.837**	**0.888**	42

The true number of identities is 38, and the total number of face images is 2,414.

**Table 4:** Comparison of clustering results in the AR Face database.

Method	F-measure	FBcubed	Clusters
ResNet-29 + *k*-means (*k*=50)	0.199	0.245	50
ResNet-29 + *k*-means (*k*=153)	0.362	0.383	153
ResNet-29 + *k*-means (*k*=239)	0.322	0.348	239
ResNet-29 + GLC	0.392	0.419	309
Approximate Rank-order [7]	—	—	—
ConPaC [4]	—	—	—
ResNet-29 + Approximate Rank-order	0.388	0.436	239
ResNet-29 + ours (proposed)	**0.447**	**0.498**	153

The true number of identities is 50, and the total number of face images is 2,600.

**Table 5:** Computation time comparison (HH : MM:SS.ms).

Method	LFW	YTF	EYaleB	AR
Number of images	13,233	621,126	2,414	2,600
ResNet-29 + *k*-means	00 : 01 : 07.663	**00** **:** **14** **:** **01.229**	**00** **:** **00** **:** **00.110**	**00** **:** **00** **:** **00.223**
ResNet-29 + GLC	**00** **:** **00** **:** **15.638**	04 : 36 : 39.981	00 : 00 : 00.276	00 : 00 : 00.289
ResNet-29 + Approximate Rank-order	00 : 04 : 06.712	03 : 26 : 34.220	00 : 00 : 29.916	00 : 00 : 33.099
ResNet-29 + ours (proposed)	00 : 00 : 16.035	04 : 41 : 31.604	00 : 00 : 00.314	00 : 00 : 00.267

## Data Availability

The Labeled Faces in the Wild data used to support the findings of this study are available at the authors' webpage at http://vis-www.cs.umass.edu/lfw/. These prior studies (and datasets) are cited at relevant places within the text as reference [13]. The YouTube Faces data used to support the findings of this study are available at the authors' web page at https://www.cs.tau.ac.il/wolf/ytfaces/. These prior studies (and datasets) are cited at relevant places within the text as reference [33]. The Extended Yale-B data used to support the findings of this study are available at the authors' web page at http://vision.ucsd.edu/iskwak/ExtYaleDatabase/ExtYaleB.html. These prior studies (and datasets) are cited at relevant places within the text as reference [11]. The AR data used to support the findings of this study are available at the authors' web page at (http://www2.ece.ohio-state.edu/~aleix/ARdatabase.html). These prior studies (and datasets) are cited at relevant places within the text as reference [34].
